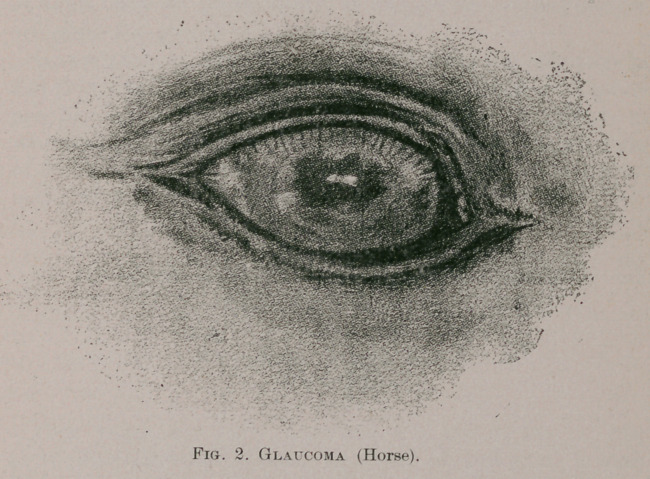# Some Rare Forms of Eye Disease in Veterinary Practice—Illustrated

**Published:** 1888-01

**Authors:** Richard W. Burke

**Affiliations:** Senior Veterinary Surgeon, Station Chief Veterinary Hospital, Jubbulpore, India


### ART. II.—SOME RARE FORMS OF EYE-DISEASE IN VETERINARY PRACTICE.

BY RICHARD W. BURKE, M. R. C. V. S., A. V. D.,
*Senior Veterinary Surgeon, Station Chief Veterinary Hospital, Jubbulpore, India.*

INTERSTITIAL KERATITIS, or so-called "salmon patch" in the eye of the horse, is not a very common affection according to my experience, and, therefore, its description may be of some importance in a clinical sense. I need not enter upon any description of the histological characteristics of the various forms of keratitis affecting the horse's eye, nor upon any discussion as to the causes supposed to engender the disease in the animal. Men may differ in their opinions as to what constitutes a "salmon patch" in the horse, as in the eye of the human subject. This condition "generally occupies the upper portion of the cornea," according to a recent writer, whilst in the case of which I give a representation in the accompanying drawing, and which was noticed in a country-bred horse belonging to the 6th Bengal Cavalry under my veterinary charge at Cawnpore, it occupied the lower division of the corneal surface (*vide* Fig. 1). In this case, the whole cornea appeared dull and opaque, and a "salmon patch" was seen covering its lower third, as shown in the above figure, for the drawing of which I am indebted to Major Radford, R. A., of Cawnpore.

*Treatment.*—The eye was shaded from the light by a bandage over the head and face, and keeping the animal in a darkened stall, and dressings of belladona and strychnine lotions were ordered, but without much benefit.

*Pathology.*—In the human subject syphilis is generally assigned as a prevailing cause, but in the horse, I think, this explanation will hardly hold good, as syphilis in the horse, excepting those cases of exceptional disease of the genitals mentioned by the French, is a unique condition, and certainly not familiar to the practitioner in England.

FIG. 1. INTERSTITIAL KERATITIS (Horse).

FIG. 2. GLAUCOMA (Horse).

GLAUCOMA.—Cases of glaucoma in the horse, although not frequent, are more common, and the accompanying drawing (see Fig. 2) is that of a case belonging to the above-named regiment, in which the condition called "contraction of the pupil" is clearly seen. In this case, the contraction is also attended by a certain "elongation" of the pupil laterally, *i. e.*, at both ends and from side to side, as seen in the figure.

*Treatment.*—A solution of eserine was ordered in this case, with more or less benefit, and continued for a fortnight or more, when I was suddenly telegraphed for to another station on temporary duty, in order to investigate and suppress an outbreak of anthrax reported as prevalent there, and subsequently to my present station, and, therefore lost sight of my patient above described. Solution of eserine, prescribed with such marked benefit in glaucoma of man, may be found useful if employed in that disease affecting the eye of the horse.

GRANULOMATA OF THE CORNEA.—A common affection in the dog, has not come under my observation in equine practice, until I saw a case a few weeks ago in a pony, in which caustic applications and the internal administration of Potas. Iodid. readily removed all trace of these non-malignant (as their cure proved) tumors.

## Figures and Tables

**Fig. 1. f1:**
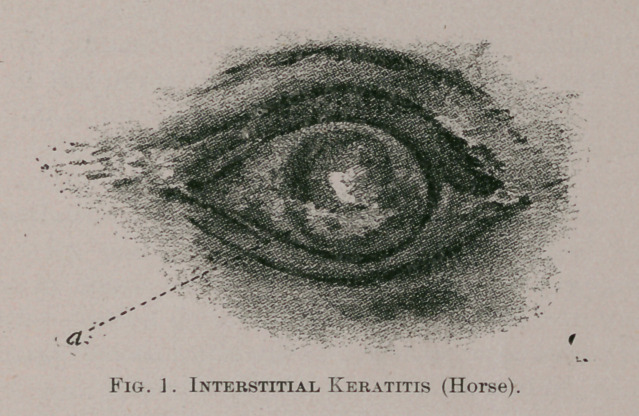


**Fig. 2. f2:**